# Shikonin-Loaded ROS-Responsive Microneedles for Psoriasis Therapy: Formulation, Transdermal Delivery, and Mechanistic Evaluation

**DOI:** 10.3390/pharmaceutics18080939

**Published:** 2026-07-30

**Authors:** Haoran Cheng, Jiaqin Dai, Lulu Cheng, Hao Liang, Yuji Zhuang, Huishan Xu, Xingxian Ou, Jun Shi

**Affiliations:** 1 School of Chinese Materia Medica, Guangdong Pharmaceutical University, Guangzhou 510006, China; 2 Guangdong Provincial Key Laboratory for Research and Evaluation of Pharmaceutical Preparations, Guangdong Pharmaceutical University, Guangzhou 510006, China

**Keywords:** shikonin, micelles, microneedles, ROS-responsive, psoriasis

## Abstract

**Background/Objectives**: Shikonin (SKN) is a potential anti-psoriatic agent, yet its clinical application is hindered by poor water solubility and low stratum corneum permeability. This study aimed to develop a reactive oxygen species (ROS)-responsive hydrogel microneedle system encapsulating SKN-loaded polymeric micelles (SKN-M@MN) to enhance transdermal delivery and evaluate its therapeutic effects in psoriasis. **Methods**: Shikonin-loaded micelles (SKN-M) were optimised using a thin-film hydration method. SKN-M@MN was fabricated via a two-step casting method using phenylboronic acid-modified hyaluronic acid (HA-PBA) and polyvinylpyrrolidone K90 as the tip matrix. Skin penetration, ROS-responsive release, and anti-psoriatic efficacy were assessed in an imiquimod (IMQ)-induced mouse model. Mechanistic studies included RNA-seq, qPCR, and Western blotting. **Results**: SKN-M achieved an encapsulation efficiency of 93.45 ± 0.24%, a particle size of 62.49 ± 0.92 nm, and a zeta potential of −36.78 ± 1.12 mV. SKN-M@MN showed 100% skin penetration, sustained drug release, and accelerated degradation under high ROS conditions. In psoriatic mice, SKN-M@MN significantly alleviated skin lesions, reduced epidermal hyperplasia (Ki67), and downregulated IL-17A and TNF-α levels both locally and systemically. Mechanistically, it inhibited the PI3K/AKT and NF-κB signalling pathways. **Conclusions**: The SKN-M@MN microneedle platform integrates physical skin penetration, ROS-responsive drug release, and pathway inhibition, offering an effective strategy for transdermal delivery of poorly soluble drugs in psoriasis therapy.

## 1. Introduction

Psoriasis is a chronic, recurrent, immune-mediated inflammatory skin disorder driven by the interplay of genetic susceptibility and environmental triggers [1,2]. It is characterised by excessive proliferation and abnormal differentiation of keratinocytes, accompanied by marked infiltration of immune cells such as T lymphocytes and dendritic cells [3,4]. Accumulating evidence indicates that oxidative stress plays a central role in the onset and progression of the disease. In psoriatic lesions, hyperactivated immune and epidermal cells generate high levels of ROS, including hydrogen peroxide and superoxide anion [5,6,7,8]. These ROS not only induce lipid peroxidation and cellular damage but also act as intracellular signalling mediators that sustain the activation of key inflammatory pathways, such as NF-κB and PI3K/AKT. This leads to increased production of pro-inflammatory cytokines, including IL-17A and TNF-α, forming a self-perpetuating “oxidative stress–inflammation” feedback loop [9,10,11]. Dysregulation of these pathways is a major driver of keratinocyte hyperproliferation and immune imbalance, underscoring the importance of targeted therapeutic strategies that can modulate oxidative stress and inflammatory signals within the disease microenvironment.

Despite advances in psoriasis treatment, current therapies remain constrained by limitations in efficacy, safety and drug delivery. Systemic treatments, such as immunosuppressants and biologics, have shown clinical efficacy in moderate-to-severe cases, but long-term use is associated with adverse effects including organ toxicity, increased infection risk and substantial economic burden [12,13,14]. Topical therapies are often preferred for their safety and direct action; however, their clinical efficacy is frequently hindered by the formidable barrier function of the stratum corneum [15,16,17]. In psoriatic lesions, hyperkeratosis further thickens this barrier, severely limiting drug penetration and leading to low bioavailability at the target site [18,19]. Therefore, overcoming the stratum corneum barrier and achieving site-specific, controlled drug release within the high-ROS microenvironment have become critical challenges in topical psoriasis therapy [20,21,22].

Shikonin, a natural naphthoquinone compound extracted from Lithospermum erythrorhizon, has attracted widespread attention owing to its potent anti-inflammatory, immunomodulatory and antioxidant activities. Previous studies have shown that SKN inhibits the NF-κB and AKT signalling pathways, reduces IL-17-induced vascular endothelial growth factor expression, and restores the expression of regulatory genes such as CCAAT enhancer binding protein delta (CEBPD). It also modulates the Th17/Treg balance by regulating the transcription factor RORγt, thereby attenuating the inflammatory cascade [23,24,25]. Moreover, SKN exhibits a selective pro-apoptotic effect on hyperproliferative keratinocytes and enhances antioxidant defence through activation of the Nuclear factor erythroid-2-related factor 2/Heme oxygenase-1(Nrf2/HO-1) pathway [26,27,28]. These multi-target effects highlight its therapeutic potential in inflammatory skin diseases, including psoriasis. However, its clinical translation is hampered by intrinsic physicochemical limitations, including extremely low water solubility, instability under light and heat, rapid systemic clearance and poor skin permeability [29,30]. These drawbacks, together with the pathological barrier of psoriatic skin, significantly limit the efficacy of conventional topical formulations of SKN.

Nanocarrier-based delivery systems offer a promising strategy to address these limitations. Polymeric micelles, particularly those formed by amphiphilic polymers such as Soluplus, have been widely used to improve the solubility and stability of hydrophobic drugs [31,32]. Soluplus exhibits a low critical micelle concentration and forms stable nanostructures with a hydrophobic core, enabling efficient encapsulation of poorly soluble compounds. Incorporation of D-α-tocopheryl polyethylene glycol 1000 succinate (TPGS) further enhances micelle stability and drug loading efficiency through synergistic effects, while also promoting cellular uptake and inhibiting drug efflux. Although such nanocarriers improve drug solubility and bioavailability, their ability to penetrate the stratum corneum remains limited when applied topically.

Microneedle technology has emerged as an effective transdermal drug delivery platform capable of overcoming the skin barrier. Composed of micron-scale needle arrays, MNs can painlessly penetrate the stratum corneum and create transient microchannels, allowing therapeutic agents to be delivered directly into the epidermis and dermis [33,34,35]. This approach combines the convenience of topical application with the efficiency of injectable administration, significantly improving drug deposition at the target site while maintaining patient compliance. Furthermore, integrating stimuli-responsive materials into MN systems enables on-demand drug release based on pathological cues.

The psoriatic microenvironment, characterised by elevated ROS levels, provides an opportunity to design ROS-responsive delivery systems [36]. In this context, HA-PBA undergoes oxidative cleavage in the presence of ROS, leading to degradation of the hydrogel matrix and triggering drug release. This mechanism not only enables site-specific controlled release but also contributes to local ROS scavenging, thereby interrupting the oxidative stress–inflammation cycle.

Given the above considerations, this study was designed to (i) optimise the preparation of SKN-loaded mixed micelles; (ii) construct an ROS-responsive HA-PBA/PVP microneedle patch; (iii) investigate its transdermal delivery performance, ROS-triggered drug release behaviour, as well as anti-psoriatic efficacy in an IMQ-induced psoriasis mouse model; and (iv) clarify the potential therapeutic mechanism associated with the PI3K/AKT/NF-κB signalling pathways. Integrating nanomicellar solubilization with microneedle-assisted transdermal penetration and ROS-responsive drug release, this multifunctional platform is developed to address the physicochemical and biological barriers that limit conventional psoriasis treatment. This innovative strategy offers a promising avenue for precise targeted drug delivery, which is conducive to improving the therapeutic efficacy of psoriasis, alleviating inflammatory lesions, and reducing off-target systemic side effects. Moreover, it provides a versatile and translatable approach for the treatment of various inflammatory skin disorders.

## 2. Materials and Methods

### 2.1. Materials

Unless otherwise stated, all chemicals and reagents were of analytical grade. The key polymers used for microneedle preparation included: Soluplus (polyvinyl caprolactam–polyvinyl acetate–polyethylene glycol graft copolymer, average Mw ≈ 118,000 g/mol, sourced from BASF (Ludwigshafen, Germany) and supplied by Shanghai Yuanye Bio-Technology Co., Ltd. (Shanghai, China), catalogue No. S35227) TPGS (D-α-tocopheryl polyethylene glycol 1000 succinate, average Mw ≈ 1513 g/mol, Shanghai Macklin Biochemical Technology Co., Ltd. (Shanghai, China), No. C15745596), PVA (polyvinyl alcohol, Mw ≈ 74,000 g/mol, Sigma-Aldrich (St. Louis, MO, USA), EFL-PVA-001, No. MKCR8876), and PVP K90 (polyvinylpyrrolidone, Mw ≈ 360,000 g/mol, Shanghai Macklin Biochemical Technology Co., Ltd., (Shanghai, China) No. C14380421). For therapeutic loading, shikonin (Chengdu Yirui Biotechnology Co., Ltd., (Chengdu, China) No. YRZ017 220701) was used as the anti-inflammatory/antioxidant agent, and HA-PBA (phenylboronic acid-modified hyaluronic acid, synthesised from hyaluronic acid with an average molecular weight of 8–14 kDa), a ROS-responsive hydrogel synthesised in our laboratory (Department of Chinese Medicine, Guangdong Pharmaceutical University, Guangzhou, China), was employed as the ROS-scavenging material.

### 2.2. Animal

A total of 40 adult female SPF SD rats (aged 6–8 weeks, body weight: 180–220 g) were obtained from Southern Medical University (Licence: SCXK (Yue) 2021-0041). The rats were raised in the SPF animal room of Guangdong Pharmaceutical University Laboratory Animal Centre, with a 12 h light-dark cycle, temperature of (24 ± 1) °C and relative humidity of (50 ± 5)%. After a 3-day adaptive feeding period, the rats were allowed ad libitum access to food and water throughout the experiment. All animal experiments complied with the regulations of the Laboratory Animal Ethics Committee of Guangdong Pharmaceutical University (Ethics No.: gdpulacspf2022382 and 14 November 2025).

### 2.3. Preparation and In Vitro Evaluation of ROS-Responsive Hydrogel Microneedles Encapsulating Shikonin-Loaded Micelles

#### 2.3.1. HPLC Analysis

Throughout the study, drug concentrations were quantified using high-performance liquid chromatography (HPLC; Thermo Fisher Scientific, Waltham, MA, USA). The HPLC system was equipped with a UV detector and an Ecosil C18 column (4.6 mm × 250 mm, 5 μm). The mobile phase consisted of 0.2% formic acid in water and acetonitrile (30:70, *v*/*v*) at an isocratic flow rate of 1.0 mL/min. The detection wavelength was 275 nm, the column temperature was maintained at 30 °C, and the injection volume was 10 μL. The HPLC method was adopted from a previously reported procedure with minor modifications [37].

#### 2.3.2. Preparation of SKN-M

SKN-M was prepared using a thin-film hydration method. Briefly, shikonin, Soluplus and TPGS were dissolved in 5 mL of anhydrous ethanol and subjected to bath sonication at 35 °C for 1 min to obtain a clear organic phase. The ethanol was then removed under reduced pressure at 40 °C using a rotary evaporator, forming a thin film on the inner wall of the flask. Subsequently, 5 mL of pre-heated distilled water was added, and the mixture was stirred magnetically at 200 rpm at the hydration temperature. The resulting crude micelle dispersion was further treated by bath sonication for 1 min to reduce particle size and polydispersity index (PDI), then filtered through a 0.45 μm membrane to obtain a clear SKN-M solution, which was stored at 4 °C protected from light.

#### 2.3.3. Characterisation of SKN-M

Freshly prepared SKN-M solution was observed under natural light for colour and clarity. In a dark room, a green laser pointer was directed laterally through the sample vial to visualise the Tyndall effect. Particle size, PDI and zeta potential were measured using a NanoBrook Omni particle size and zeta potential analyser (Brookhaven Instruments Corporation, Holtsville, NY, USA) at 25 °C. For Dynamic light scattering (DLS) measurements, a solid-state laser with a wavelength of 660 nm was used as the light source, and scattered light was detected at a fixed angle of 90°. Prior to measurement, each sample was equilibrated at 25 °C for 2 min to ensure thermal stability. The intensity autocorrelation function was acquired and analysed using the cumulants method to obtain the Z-average hydrodynamic diameter (intensity-weighted mean) and the PDI. Each sample was measured in triplicate, and the reported particle sizes correspond to the intensity-weighted hydrodynamic diameter unless otherwise specified.

The entrapment efficiency (EE) of SKN-M was determined by a centrifugation method combined with HPLC analysis. 2 mL of the SKN-M solution was centrifuged at 4000 rpm for 10 min to precipitate unencapsulated drug crystals. The supernatant (1 mL) was carefully transferred to a 5 mL volumetric flask, mixed with methanol, and bath-sonicated at 30 °C for 10 min to break the micelles. After cooling to room temperature, the solution was brought to the mark with methanol (final volume 5 mL) and mixed well. The resulting solution was filtered through a 0.45 μm membrane, and the filtrate was collected for HPLC analysis. The measured drug concentration was recorded as C_1_ (encapsulated drug concentration).

Separately, 1 mL of uncentrifuged SKN-M stock solution was directly transferred to a 5 mL volumetric flask and subjected to the same procedure (methanol addition, bath sonication at 30 °C for 10 min, cooling, dilution to volume, and filtration) followed by HPLC analysis to determine the total drug concentration, recorded as C_2_. The entrapment efficiency was calculated using Equation (1):

(1) $${\mathrm{EE}}_{\mathrm{SKN}}\%\;=\;\frac{C_{1}}{C_{2}}\;\times \;100\%$$

#### 2.3.4. Optimisation of SKN-M Preparation Parameters

Based on the characterisation results, single-factor experiments were conducted using entrapment efficiency (EE) as the primary evaluation index. In these single-factor experiments, only one parameter was varied at a time, while all other parameters were fixed at the baseline conditions (Soluplus:TPGS = 1.5:1, shikonin:total excipients = 1:40, hydration temperature = 40 °C, and hydration time = 40 min). The four factors investigated were: mass ratio of Soluplus to TPGS (0.5:1 to 3.5:1), mass ratio of total excipients to shikonin (1:20 to 1:60), hydration temperature (35 °C to 55 °C), and hydration time (20 to 60 min). For each factor, the optimal level was identified based on the highest EE obtained while maintaining the other parameters at the baseline values. Three independent batches were prepared per condition.

#### 2.3.5. Preparation of SKN-M@MN

SKN-M solution prepared under the optimal conditions (determined in Section 2.3.4) was pre-frozen at −20 °C and then lyophilised to obtain shikonin micelle lyophilised powder.

HA-PBA was synthesised using the DMT-MM coupling method. HA (0.5 g) was dissolved in water/DMF (3:2, *v*/*v*), and DMT-MM (0.346 g, 1.25 mmol) was added to activate for 30 min. Then, 3-aminophenylboronic acid (0.137 g, 1.0 mmol) was added, and the pH was adjusted to 6.5 with NaOH. The reaction was allowed to proceed at room temperature for 24 h. The resulting solution was dialyzed against deionized water using dialysis tubing with a molecular weight cutoff (MWCO) of 8–14 kDa for 3 days, followed by lyophilization to yield HA-PBA.

Microneedles were fabricated using a two-step casting method. The mould had a needle height of 500 μm and a diameter of 200 μm. HA-PBA and PVP K90 (mass ratio 1:2) were dissolved in distilled water, and a predetermined amount of SKN-M lyophilised powder was added, followed by sonication in the dark to obtain a homogeneous drug-loaded tip matrix solution. The solution was introduced into the mould, and centrifuged at 4000 rpm for 10 min to ensure complete filling of the microcavities. A backing layer solution (1 g PVP-K90 and 1 g PVA dissolved in 5 mL distilled water, degassed by sonication) was then added and centrifuged at 4000 rpm for 5 min, followed by drying at 37 °C for 12 h. After complete solidification, the microneedle patches were carefully peeled off and stored in a dry container protected from light.

#### 2.3.6. Characterisation of SKN-M@MN

The appearance of SKN-M@MN patches prepared under the optimal conditions was observed with the naked eye and by scanning electron microscopy (SEM) to examine the arrangement of the microneedle array, needle sharpness, and the presence of structural defects (e.g., collapse, bubbles, microcracks) on the needle and backing layer surfaces, thereby providing a comprehensive assessment of moulding quality.

#### 2.3.7. Optimisation of SKN-M@MN Formulation

To optimise the mass ratio of tip matrix to micelle lyophilised powder, microneedles were prepared at ratios of 1:1.5, 1:2, 1:2.5 and 1:3.5, and evaluated for mouldability, skin penetration rate and mechanical performance.

Mouldability: The integrity of the needles and the number of needles in the array were examined under a microscope.

Skin penetration rate: Rat dorsal skin was excised, depilated, and the subcutaneous fat was removed. The skin was cleaned with physiological saline and stored at −20 °C. Before the experiment, the skin was thawed and mounted with the stratum corneum side up. The microneedle patch was pressed vertically onto the skin for 2 min, followed by staining with 0.5% methylene blue for 1 min. Excess stain was removed with isopropanol, and the percentage of blue micro-holes was calculated as Equation (2):

(2) $$\mathrm{Skin}\;\mathrm{penetration}\;\mathrm{rate}\;=\frac{\mathrm{Number}\;\mathrm{of}\;\mathrm{blue}\;\mathrm{microholes}}{\mathrm{Total}\;\mathrm{number}\;\mathrm{of}\;\mathrm{microneedles}}\times \;100\%$$

Mechanical strength: An aluminium foil sheet (approximately 24 μm thick per layer) was spread flat, and the microneedle patch was placed on the foil. A consistent compressive force was applied manually by thumb pressure for 30 s to ensure full contact between the patch and the foil surface, and the porosity through four layers of aluminium foil was observed. This aluminium foil penetration assay served as a qualitative screening method to compare the relative mechanical integrity of microneedle patches prepared with different formulation ratios, rather than to determine absolute mechanical parameters.

The three indicators (needle integrity rate, skin blue-hole rate and aluminium foil porosity) were scored as follows: 100 points for 100%, 90 points for 90%, 80 points for 80%, and so on.

At all four examined mass ratios, the mouldability score was 100 points. For skin penetration rate and mechanical performance, mass ratios of 1:1.5, 1:2 and 1:2.5 all achieved 100 points. However, when the ratio was increased to 1:3.5, the skin penetration rate decreased to 80 points and the mechanical performance score dropped to 70 points. Therefore, a mass ratio of 1:2.5 was selected as the optimal formulation, providing both excellent performance and sufficient drug loading capacity.

#### 2.3.8. Microneedle Drug Loading Determination

Three batches of SKN-M@MN patches were randomly selected. The needle tips were cut off and placed in 5 mL beakers containing a mixture of distilled water and methanol, then bath-sonicated for 20 min to dissolve the tip matrix and break the micelles. The resulting solution was filtered through a 0.45 μm membrane, and the shikonin content was determined by HPLC to calculate the actual drug loading per patch.

#### 2.3.9. Characterisation of Micellar Properties of Drug-Containing Microneedles After Reconstitution

To evaluate the structural stability of SKN-M during microneedle matrix preparation and lyophilisation, an appropriate number of patches were completely dissolved in distilled water. The resulting solution was appropriately diluted, and the average particle size and PDI (intensity-weighted Z-average) were measured and compared with the data of the original SKN-M solution before lyophilisation to verify the integrity of the nanostructures. A drop of the diluted reconstituted solution was also placed on a transmission electron microscope (TEM) grid to observe the morphology.

#### 2.3.10. Skin Irritation Test

Healthy SD rats were depilated on both sides of the dorsal spine (approximately 5 cm × 5 cm per side) 24 h before the experiment. The left depilated area was treated with pH 7.4 phosphate-buffered saline (PBS) as a self-control. The rats were randomly divided into four groups, and the right depilated area received the following treatments: (1) blank control: physiological saline; (2) positive control: formaldehyde solution; (3) micelle group: SKN-M solution; (4) microneedle group: SKN-M@MN patch pressed vertically for 2 min and fixed with medical tape. After 24 h, the rats were euthanised, and the treated skin was excised, fixed in 4% paraformaldehyde, embedded in paraffin, sectioned, and stained with haematoxylin and eosin (H&E) for light microscopy observation.

#### 2.3.11. In Vitro Release Test

To characterise the fundamental drug release behaviour of SKN-M before evaluating its skin permeation and in vivo performance, the in vitro release test was carried out using a dialysis bag method. SKN-M solution (3 mL) and an equivalent amount of free shikonin suspension (as control) were each placed into pre-treated dialysis bags (MWCO 8000–14,000 Da), with three replicates per group. The sealed bags were submerged in 150 mL of release medium (pH 6.8 PBS containing 0.5% Tween-80) under light-protected conditions. The setup was shaken at 37 °C and 100 rpm. At predetermined time points (0.5, 1, 2, 4, 6, 8, 12, 24 and 48 h), 1 mL of release medium was withdrawn and immediately replaced with an equal volume of fresh pre-warmed medium. The withdrawn samples were centrifuged at 12,000 rpm for 10 min, and the supernatant was analysed by HPLC. The cumulative release percentage was calculated using Equation (3), and release profiles were plotted.

(3) $$Q_{n}=\frac{C_{n}\;\times \;V+\sum_{i=0}^{n-1}C_{i}\times \;V_{i}}{W}\times \;100\%$$

where C_n_ is the drug concentration in the sample at the nth time point, V is the total volume of release medium, C_i_ is the drug concentration in the sample at the ith time point, V_i_ is the sampling volume, and W is the total amount of drug initially placed in the dialysis bag.

#### 2.3.12. In Vitro Skin Permeation Study

Rat dorsal skin previously stored at −20 °C was thawed at room temperature for 30 min and rinsed with physiological saline to remove residual blood and debris. The subcutaneous fat and connective tissue were carefully removed using surgical scissors and a scalpel, leaving full-thickness skin (epidermis and dermis) with an intact stratum corneum. The skin thickness after removal of subcutaneous fat was approximately 0.4–0.9 mm, consistent with previously reported values for SD rat dorsal skin [38]. The skin was then cut into appropriately sized pieces and soaked in physiological saline at room temperature for approximately 30 min to rehydrate the tissue prior to use. The integrity of the stratum corneum was verified by visual inspection and by measuring the trans-epidermal electrical resistance; only skin samples with resistance values above 10 kΩ were used for permeation experiments. The pre-treated skin was mounted between the donor and receptor chambers of a Franz diffusion cell with the stratum corneum facing the donor chamber. The receptor chamber was filled with 14 mL of PBS (pH 7.4) containing a small amount of surfactant, maintained at 37 °C ± 0.5 °C by a water jacket, and stirred magnetically at 100 rpm to simulate blood microcirculation.

Three groups were tested: free SKN solution, SKN-M, and SKN-M@MN. The microneedle patch was pressed vertically onto the skin centre with moderate pressure for 2 min to ensure needle penetration, then fixed in place. At predetermined time points (0.5, 1, 2, 4, 6, 8, 10, 12, 24, 36 and 48 h), 1 mL of receptor fluid was withdrawn and immediately replaced with an equal volume of fresh receptor fluid. Shikonin concentration in the withdrawn samples was determined by HPLC, and the cumulative amount permeated per square centimetre was calculated using Equation (4):

(4) $$Q_{n}=\frac{C_{n}\;\times \;V+\sum_{i=0}^{n-1}C_{i}\times \;V_{i}}{A}\;\times \;100\%$$

where C_n_ is the drug concentration in the sample at the nth time point, C_i_ is the drug concentration at the ith (i = n − 1) time point, V is the receptor volume (14 mL), V_i_ is the sampling volume, and A is the effective diffusion area (1.766 cm^2^).

#### 2.3.13. Cell Compatibility Evaluation

The CCK-8 assay was used to evaluate the effect of different formulations on the viability of human epidermal keratinocytes (HaCaT cells). HaCaT cells in the logarithmic growth phase were seeded into 96-well plates at a density of 5 × 10^3^ cells/well and incubated at 37 °C in 5% CO_2_ for 24 h to allow full attachment. Cells were then treated with blank SKN-M@MN extract or blank SKN-M extract, with a cell control group (cells with normal culture medium). After further incubation for 24 h, 10 μL of CCK-8 reagent was added to each well in the dark, and the plates were incubated for another 2 h. The absorbance (OD value) at 450 nm was measured using a microplate reader, and cell viability was calculated to assess biocompatibility.

#### 2.3.14. Cell Uptake Experiment and CLSM Fluorescence Observation

Coumarin-6 (C6) was used as a fluorescent surrogate for shikonin to prepare C6-labelled micelles and microneedles (C6-M@MN). HaCaT cells were seeded into 6-well plates containing sterile glass coverslips at a density of 1.1 × 10^5^ cells/well and incubated for 24 h. The cells were then treated with fresh medium containing free C6, C6-M or C6-M@MN extract (final C6 concentration 3 μmol/L) for 2 h. After incubation, the cells were washed three times with cold PBS, fixed with 4% paraformaldehyde for 15 min, and the nuclei were counterstained with 4′,6-Diamidino-2-phenylindole(DAPI) for 15 min. After washing and mounting, the samples were observed and imaged using a confocal laser scanning microscope (CLSM).

#### 2.3.15. In Vitro Evaluation of ROS-Triggered Drug Release from SKN-M@MN

The release medium was pH 6.8 PBS. For the low-ROS group, blank PBS was used; for the high-ROS group, H_2_O_2_ was added to a final concentration of 1.0 mmol/L. Ten millilitres of each medium was placed in a glass sample vial and pre-warmed at 37 °C with shaking at 100 rpm. Intact SKN-M@MN patches with consistent drug loading were submerged in the corresponding release medium and incubated in the dark protected from light. At 0.5, 1, 2, 4, 8, 12, 24 and 36 h, 1.0 mL of release medium was withdrawn and replaced with an equal volume of fresh pre-warmed medium of the same composition. The withdrawn samples were centrifuged at 12,000 rpm for 10 min, and the supernatant was filtered through a 0.45 μm membrane. Shikonin concentration was determined by HPLC. The cumulative release percentage was calculated based on the absolute drug loading per microneedle patch, and release curves were plotted to compare the release profiles between low- and high-ROS environments.

### 2.4. Animal Experiments

**Figure 1:**
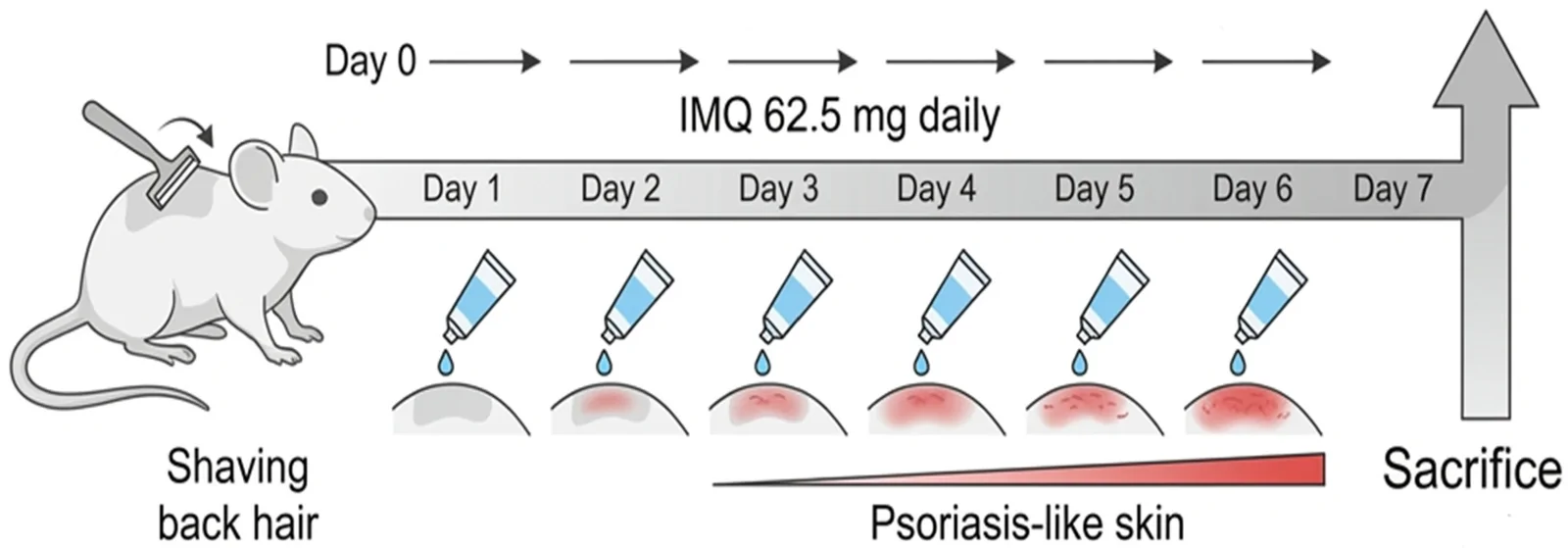
Method for modelling psoriasis in mice.

The psoriasis model and treatment regimen are illustrated in Figure 1. An IMQ-induced psoriasis-like dermatitis model was established in female BALB/c mice (aged 6–8 weeks, weighing 18–22 g, *n* = 6 per group) [39]. Psoriasis-like skin lesions were induced by daily topical application of 5% imiquimod (IMQ) cream (62.5 mg per mouse, containing 3.125 mg of IMQ) to the shaved dorsal skin for 7 consecutive days.

The mice were randomly divided into six groups (*n* = 6 per group): control group (shaved skin only, no treatment), model group (IMQ only), positive control group (IMQ + 0.05% dexamethasone cream, 25 mg per mouse), free SKN group (IMQ + topical shikonin solution, 200 μg shikonin in 100 μL PBS per mouse), SKN-M group (IMQ + topical SKN-M solution, 200 μg shikonin equivalents in 100 μL PBS per mouse), and SKN-M@MN group (IMQ + SKN-M@MN patch applied with manual pressure for 2 min). Each group received daily treatment once daily for 7 consecutive days. All treatments were administered at approximately the same time each day (9:00–10:00 AM) to minimise circadian variability.

The SKN-M@MN patches used in the animal study measured 1 cm × 1 cm, containing a 10 × 10 array of microneedles (total 100 needles per patch, needle height 500 μm, base diameter 200 μm, tip diameter < 10 μm). Each patch contained 252.07 ± 1.89 μg of shikonin. To standardise the application procedure, the patch was pressed manually against the shaved dorsal skin using the thumb for 2 min. The pressure was visually standardised by ensuring the patch backing layer was fully in contact with the skin surface and the microneedles were completely embedded; the same investigator performed all applications to maintain consistency across animals.

Psoriasis Area and Severity Index (PASI) score: Back skin lesions were scored daily based on erythema, scaling and thickness (0–4 scale).

Spleen index: The ratio of spleen weight (mg) to body weight (g) was calculated.

Cytokine detection: The levels of Tumour Necrosis Factor-alpha (TNF-α), Interleukin-6 (IL-6) and interleukin 17A (IL-17A) in serum and skin homogenates were quantitatively measured using Enzyme-Linked Immunosorbent Assay (ELISA) kits according to the manufacturer’s instructions.

Histology and immunohistochemistry: Skin sections (4–5 μm thick) were stained with H&E, and epidermal thickness was measured using ImageJ software (version 1.54f, National Institutes of Health, Bethesda, MD, USA) (five regions per section). For immunohistochemistry, sections were incubated with anti-Ki67 antibody (dilution 1:200, overnight at 4 °C), followed by horseradish peroxidase (HRP)-conjugated secondary antibody, and finally visualised with diaminobenzidine (DAB).

RT-qPCR: Total RNA was extracted using TRIzol and cDNA was synthesised using a reverse transcription kit. qPCR was performed using SYBR Green under the following conditions: 95 °C for 30 s, followed by 40 cycles of 95 °C for 5 s and 60 °C for 30 s. Primers for TNF-α, IL-6, interleukin-23 (IL-23), IL-17A and glyceraldehyde-3-phosphate dehydrogenase (GAPDH) were used, and relative expression levels were calculated using the 2^−ΔΔCt^ method.

Western blotting: Skin tissue was lysed in RIPA buffer containing protease and phosphatase inhibitors. Equal amounts of protein were subjected to sodium dodecyl sulphate polyacrylamide gel electrophoresis (SDS-PAGE), transferred to Polyvinylidene Fluoride (PVDF) membranes, blocked with 5% non-fat milk, and incubated with primary antibodies (anti-PI3K, p-AKT, AKT and GAPDH, all at 1:1000 dilution, overnight at 4 °C), followed by HRP-conjugated secondary antibodies (1:5000 dilution, 1 h). Signals were visualised using ECL and quantified with ImageJ software (version 1.54f, National Institutes of Health, Bethesda, MD, USA) (five regions per sec-tion).

RNA-seq: Total RNA was extracted, libraries were constructed, and sequencing was performed on an Illumina platform. Raw reads were trimmed and aligned to the mouse reference genome. Differential expression analysis was performed using DESeq2, and heatmaps were generated using the R package seaborn.

### 2.5. Statistical Analysis

Statistical analysis and graphical visualisation were performed using GraphPad Prism version 8.3.0. To ensure accuracy and reliability, each experiment was repeated at least three times (biological replicates) to minimise random error. All data are presented as mean ± SEM. Comparisons between two groups were made using independent samples t-tests, and among multiple groups using one-way analysis of variance (ANOVA), as appropriate. A *p*-value ≤ 0.05 was considered statistically significant.

## 3. Results

### 3.1. Preparation of SKN-M

SKN-M was prepared using a thin-film hydration method as described in Section 2.3.2. The resulting SKN-M solution appeared homogeneous and clear, with a light purplish-red colour, indicating successful encapsulation of shikonin into the polymeric micelles.

### 3.2. Optimisation of SKN-M Preparation Parameters

To identify the optimal preparation conditions, single-factor experiments were conducted using entrapment efficiency (EE) as the primary evaluation index. Four factors were investigated: the mass ratio of Soluplus to TPGS, the mass ratio of total excipients to shikonin, the hydration temperature and the hydration time. As shown in Table 1, the EE first increased and then decreased with increasing values of each of these four parameters. The optimal level for each factor was determined to be: Soluplus:TPGS mass ratio of 1.5:1 (EE = 92.94%), shikonin:excipients mass ratio of 1:40 (EE = 91.86%), hydration temperature of 45 °C (EE = 92.31%), and hydration time of 50 min (EE = 93.46%).

The optimal combination (Soluplus:TPGS = 1.5:1, shikonin:excipients = 1:40, hydration temperature = 45 °C, hydration time = 50 min) was confirmed by triplicate verification. Three independent batches of SKN-M prepared under these conditions yielded EE values of 93.52%, 93.18%, and 93.65%, with an average of 93.45 ± 0.24%. These results demonstrate that the optimised formulation process is stable, feasible, and highly reproducible, enabling efficient and stable encapsulation of shikonin.

### 3.3. Characterisation of SKN-M

**Figure 2:**
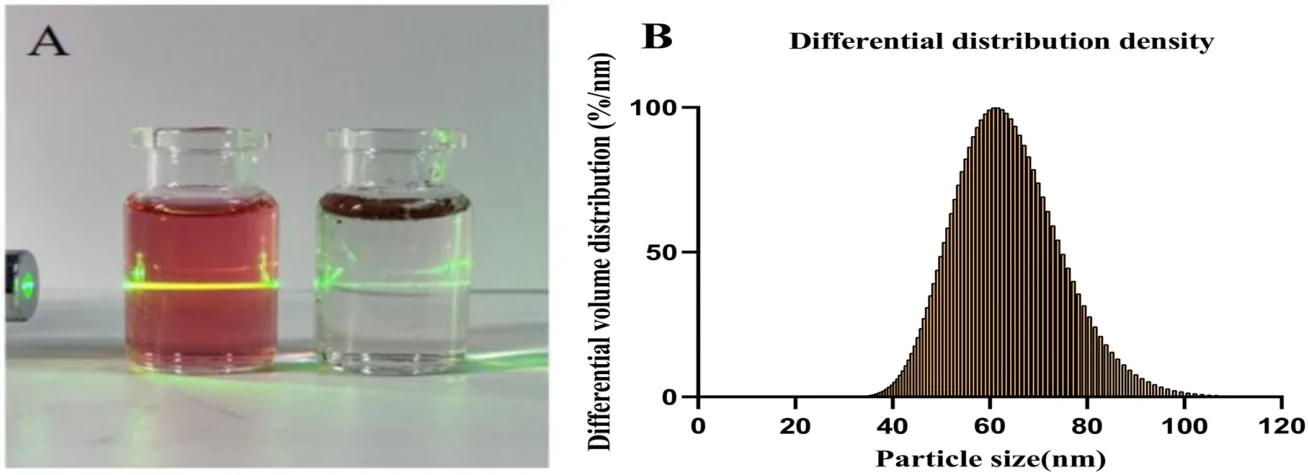
(**A**) Appearance and Tyndall effect, (**B**) particle size distribution.

SKN-M prepared under the optimal conditions was further characterised for its physicochemical properties. As shown in Figure 2A, the SKN-M solution appeared homogeneous, clear and light purplish-red. When a green laser beam was directed laterally through the sample vial, a clear Tyndall effect light path was observed within the micelle solution. In contrast, in the shikonin-in-water control group, the insoluble drug remained as solid particles floating on the liquid surface, and no continuous Tyndall light path was observed under laser illumination.

DLS measurements (Figure 2B) showed that the size distribution of SKN-M exhibited a single unimodal peak, with an intensity-weighted average hydrodynamic diameter (Z-average) of 62.49 ± 0.92 nm and a polydispersity index (PDI) of 0.127 ± 0.012. The surface charge measurement revealed an average zeta potential of −36.78 ± 1.12 mV for the mixed micelle system.

As shown in Table 2, SKN-M remained physically stable for 14 days when stored at room temperature and at 0–4 °C, with no precipitation or turbidity. The particle size increased slightly from 62.49 nm to 70.24 nm (room temperature) and to 66.89 nm (0–4 °C). The PDI values remained below 0.148 under all conditions, indicating good storage stability.

### 3.4. Preparation of SKN-M@MN

SKN-M@MN was fabricated using a two-step casting method as described in Section 2.3.5. The resulting microneedle patches exhibited uniform light purplish-red colour, indicating successful loading of the drug-loaded micelles into the microneedle matrix.

### 3.5. Optimisation of SKN-M@MN Formulation

To optimise the mass ratio of tip matrix to micelle lyophilised powder, microneedles were prepared at ratios of 1:1.5, 1:2, 1:2.5 and 1:3.5, and evaluated for mouldability, skin penetration rate and mechanical performance. At all four examined mass ratios, the mouldability score was 100 points. For skin penetration rate and mechanical performance, mass ratios of 1:1.5, 1:2 and 1:2.5 all achieved 100 points. However, when the ratio was increased to 1:3.5, the skin penetration rate decreased to 80 points and the mechanical performance score dropped to 70 points. Therefore, a mass ratio of 1:2.5 was selected as the optimal formulation, providing both excellent performance and sufficient drug loading capacity. The detailed scoring data are provided in Table S1 (Supplementary Information).

### 3.6. Characterisation of SKN-M@MN

**Figure 3:**
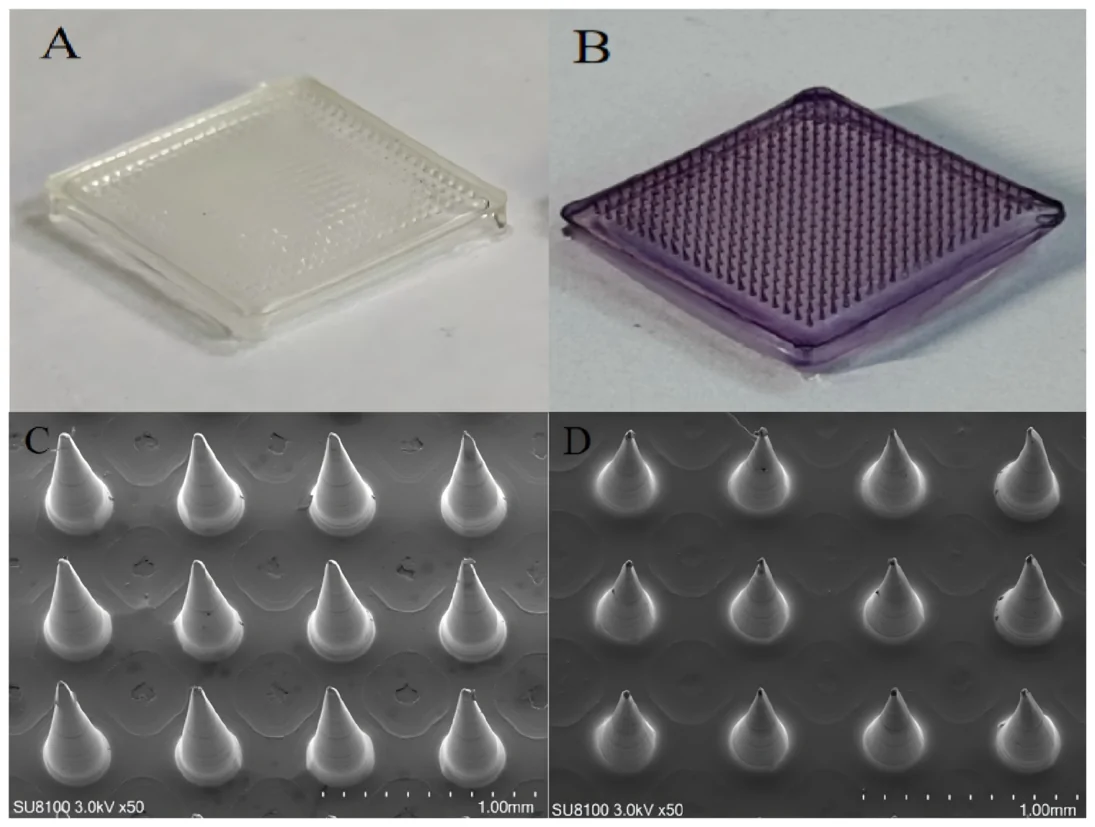
(**A**) Macroscopic photographs of blank microneedles; (**B**) Macroscopic photographs of drug-loaded microneedles; (**C**) SEM images of blank microneedles; (**D**) SEM images of drug-loaded microneedles.

SKN-M@MN prepared under the optimal conditions (mass ratio of 1:2.5) was further characterised. As shown in Figure 3A, the blank hydrogel microneedles appeared colourless and transparent. The shikonin-loaded microneedle patches (Figure 3B) exhibited a uniform light purplish-red colour. Scanning electron microscopy (SEM) images showed that both blank microneedles (Figure 3C) and drug-loaded microneedles (Figure 3D) presented well-ordered arrays, regular conical needle bodies with essentially uniform height (needle length 600 μm, pitch 1000 μm, sharp tips, base diameter 250 μm, array size 10 × 10). Moreover, the drug-loaded microneedles exhibited smooth needle surfaces and a flat interface with the backing layer, with no obvious collapse, bubbles, microcracks or precipitation of free drug particles. The average absolute shikonin loading per patch was 252.07 ± 1.89 μg (*n* = 3), indicating stable drug loading and good inter-patch uniformity.

### 3.7. Characterisation of Micellar Properties of Drug-Containing Microneedles After Reconstitution

**Figure 4:**
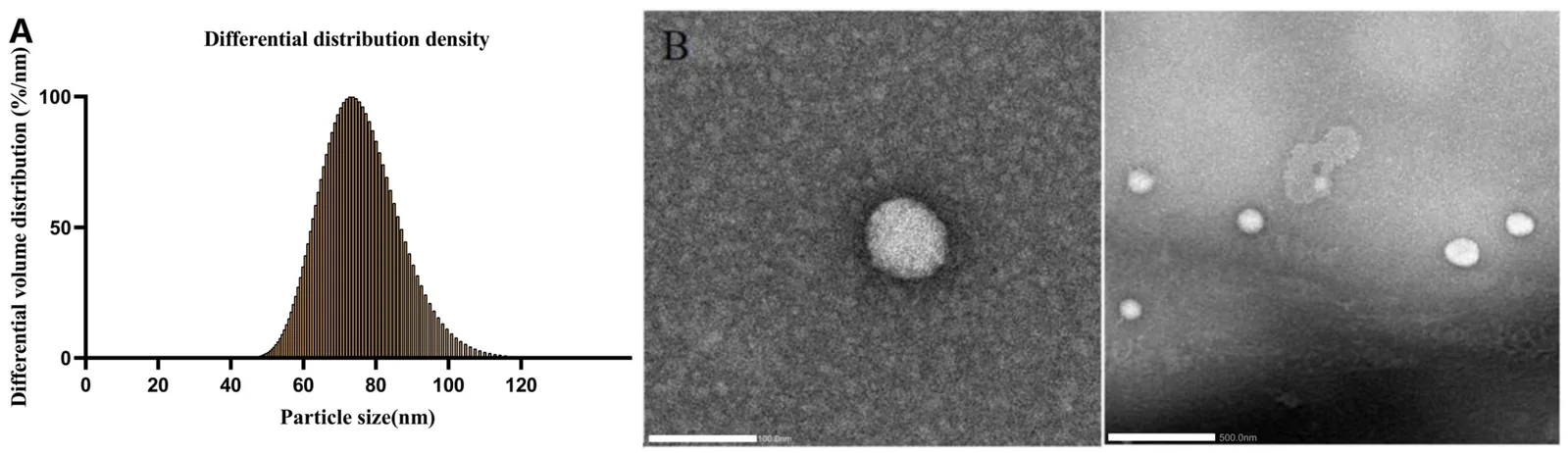
(**A**) Characterisation of reconstituted micelles: DLS size distribution of reconstituted micelles; (**B**) TEM images of reconstituted micelles. The scale bar represents 100 nm.

To examine the structural stability of SKN-M during microneedle matrix embedding and solidification, the completely reconstituted solution of SKN-M@MN was characterised by DLS and TEM, and the results are presented in Figure 4. DLS measurements (Figure 4A) showed that after reconstitution, SKN-M still exhibited a regular unimodal size distribution, with an intensity-weighted average hydrodynamic diameter (Z-average) of 73.67 ± 1.12 nm, a PDI of 0.135 ± 0.019 and a zeta potential of −30.67 ± 1.66 mV. Compared with the original micelles before lyophilisation, the particle size and distribution of the reconstituted micelles showed no significant changes, and the system maintained a good monodisperse state.

TEM images (Figure 4B) further confirmed the integrity of the microstructure. The reconstituted micelles in the field of view still exhibited regular spherical or spheroidal nanostructures with clear edges. The particles were dispersed independently, with no obvious adhesion, fusion, emulsion breaking or precipitation of free drug crystals caused by lyophilisation or physical compression from the matrix.

### 3.8. Biocompatibility and Cellular Uptake

**Figure 5:**
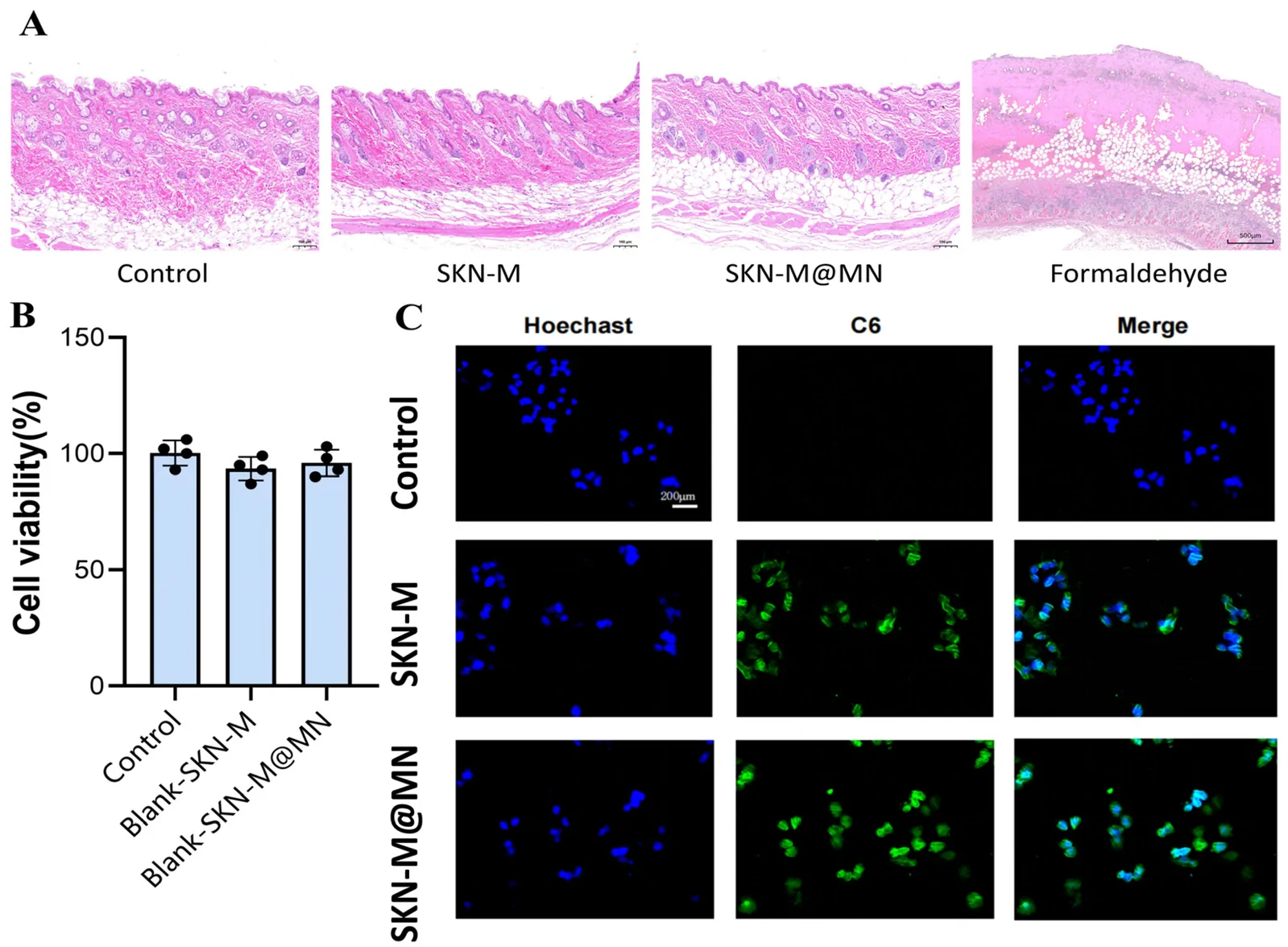
(**A**) H&E staining of skin sections treated with Control, SKN-M, SKN-M@MN, and formaldehyde (×40). Hematoxylin stains cell nuclei in blue-purple, and eosin stains the cytoplasm in pink. (**B**) Cell viability detected by CCK-8 assay. (**C**) Representative fluorescence microscopy images of HaCaT cells after uptake (×400). Hoechst 33342 stains cell nuclei in blue (left panel), SKN-M micelles show green fluorescence (middle panel), and the merged images (right panel) demonstrate the co-localization of SKN-M with the cells.

As shown in Figure 5A, application of SKN-M and SKN-M@MN to the skin caused a slight, transient erythema immediately after microneedle insertion, which subsided within 2 h, with the skin returning to its normal state. The irritation scores and H&E staining results indicated that no skin barrier damage, infection or inflammatory response was observed after application of SKN-M or SKN-M@MN on the backs of rats. These findings confirm that both SKN-M and SKN-M@MN possess favourable skin safety and biocompatibility.

The CCK-8 assay was used to evaluate the biocompatibility of the blank carriers and the microneedle matrix. As shown in Figure 5B, the viability of HaCaT cells incubated with extracts of blank SKN-M@MN or blank SKN-M was above 90%, indicating that the Soluplus/TPGS carriers and the microneedle matrix have good biosafety and show no significant cytotoxicity.

Cellular uptake was visualised by confocal laser scanning microscopy (CLSM). As shown in Figure 5C, both the C6/SKN-M@MN and C6/SKN-M groups exhibited strong and dense green fluorescence in the cytoplasm of HaCaT cells. In contrast, the free C6 control group showed extremely weak fluorescence. These results demonstrate that the SKN-M@MN delivery system effectively promotes the uptake of the model drug by keratinocytes.

### 3.9. In Vitro and In Vivo Anti-Psoriatic Efficacy

**Figure 6:**
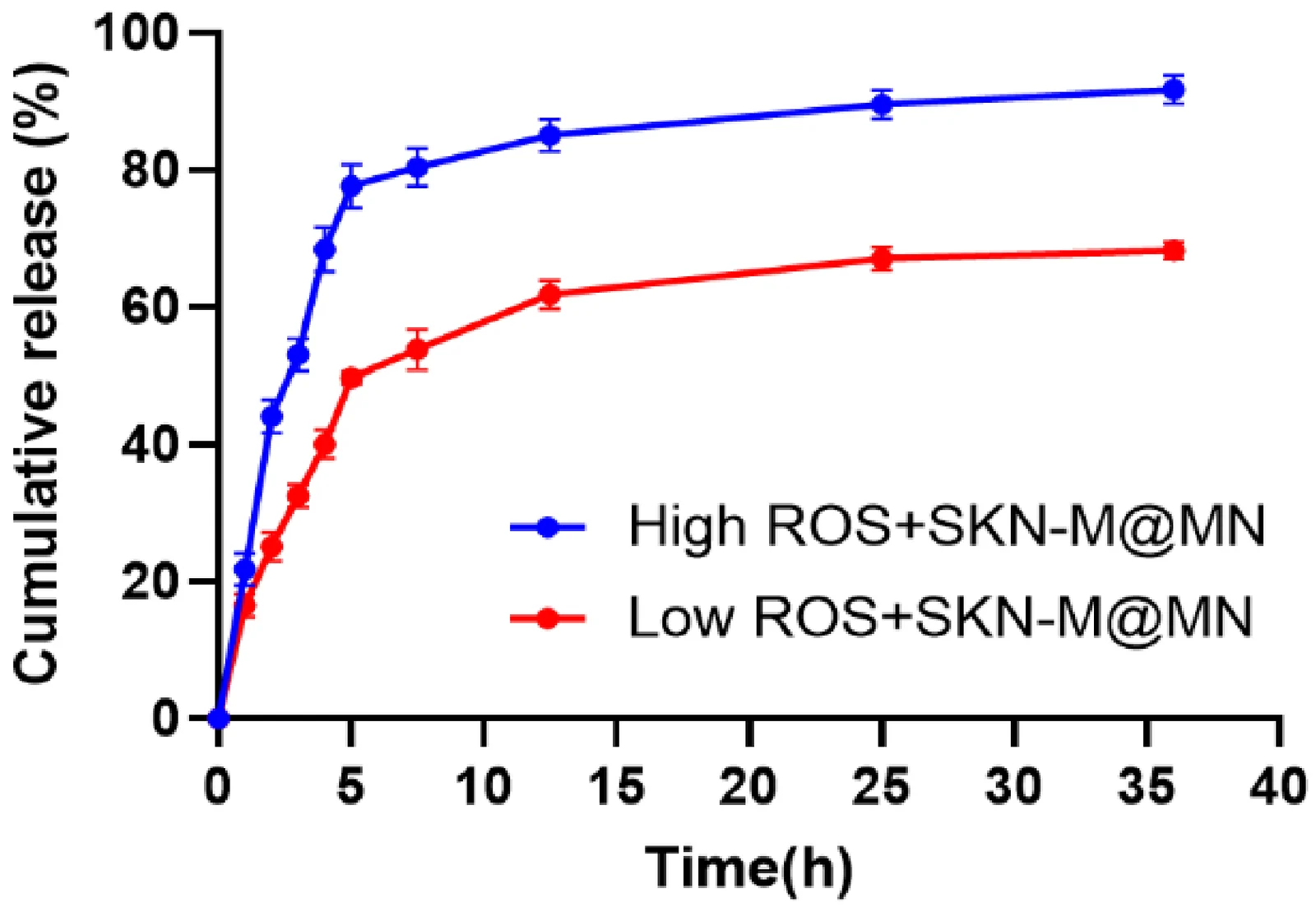
ROS-responsive in vitro release curve.

As shown in Figure 6, compared with the low-ROS environment, SKN-M@MN exhibited a faster shikonin release rate and a higher cumulative release in the high-ROS environment.

**Figure 7:**
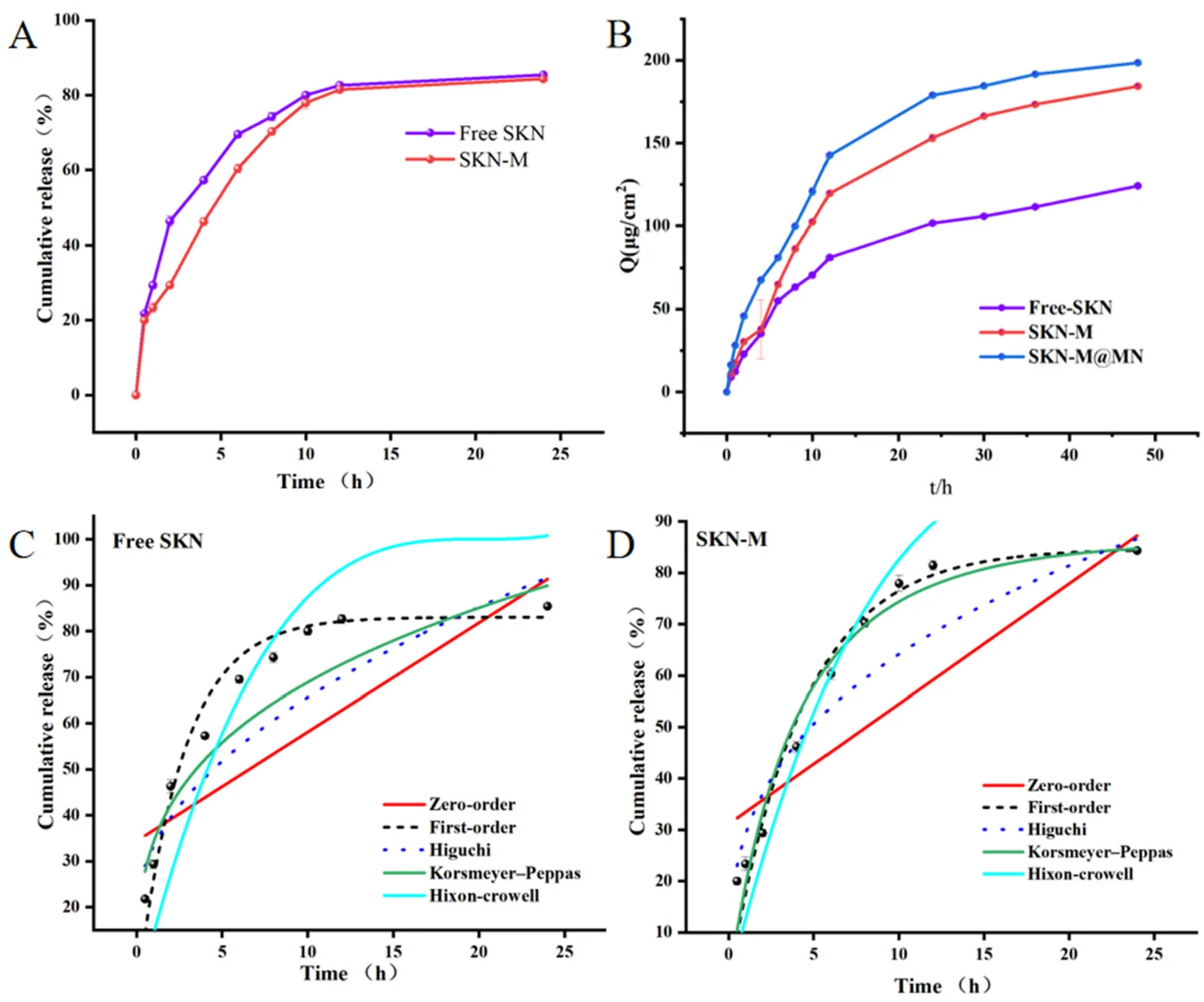
(**A**) In vitro release profiles of Free SKN and SKN-M; (**B**) skin permeation profiles of Free SKN, SKN-M, and SKN-M@MN; (**C**,**D**) drug release kinetic fitting curves of Free SKN and SKN-M.

The in vitro cumulative release profiles of free shikonin (Free-SKN) and shikonin-loaded micelles (SKN-M) are presented in Figure 7A. During the initial release phase (first 6 h), the Free-SKN group showed a rapid release rate, with cumulative release reaching approximately 70%; in contrast, the SKN-M group exhibited a relatively slower release, with cumulative release around 60% at 6 h. Over time, both groups essentially reached a release plateau within 24 h (cumulative release > 80%).

As shown in Figure 7B, the cumulative permeation of all three formulations increased over time, but significant differences were observed in the permeation rate and total cumulative amount. Throughout the entire observation period, the order of cumulative permeation was consistently SKN-M@MN > SKN-M > Free-SKN. The Free-SKN group exhibited the lowest permeation curve, with the slowest overall permeation rate and the lowest final cumulative amount at 48 h. The SKN-M group showed a markedly higher permeation curve than the free drug group, with significantly increased cumulative amounts at all time points. The SKN-M@MN group exhibited the highest permeation curve, with the steepest increase during the first 12 h (i.e., a faster initial permeation rate). At the end of the experiment, the cumulative permeation of this group remained higher than that of both the Free-SKN and SKN-M groups.

To further explore the release mechanism, the release data were fitted to several classical kinetic models, and the results are summarised in Table 3. The release behaviour of both Free-SKN and SKN-M was best described by the first-order kinetic model (Figure 7C,D). The kinetic fitting indicated that shikonin release from the mixed micelles was concentration-dependent. Moreover, the first-order release rate constant of SKN-M (k = 0.2333) was lower than that of free shikonin (k = 0.3751), further confirming the sustained-release characteristics of the micelle system.

### 3.10. Animal Experiments

**Figure 8:**
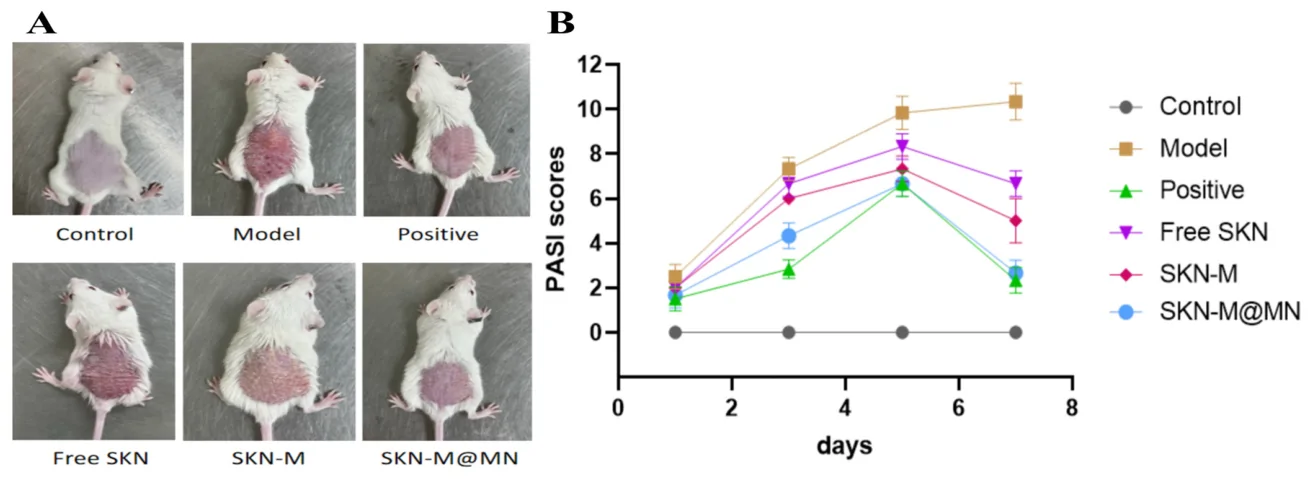
(**A**) Skin lesions on the back of mice in each group on day 7 and corresponding PASI scores; (**B**) Trend plot of PASI scores for dorsal condition in each group (*n* = 6).

The macroscopic appearance of the back skin on day 7 is shown in Figure 8A. Model mice exhibited typical psoriasis-like lesions (erythema, scaling, thickening), and all treatments alleviated these symptoms. SKN-M@MN almost completely resolved erythema and scaling, showing the best improvement, although mild roughness remained. Figure 8B shows the PASI scores over 7 days. All treatment groups showed a peak on day 5 followed by a decline. On day 7, both the positive control and SKN-M@MN significantly reduced the PASI score compared with the model group, whereas Free SKN and SKN-M showed only modest effects.

**Figure 9:**
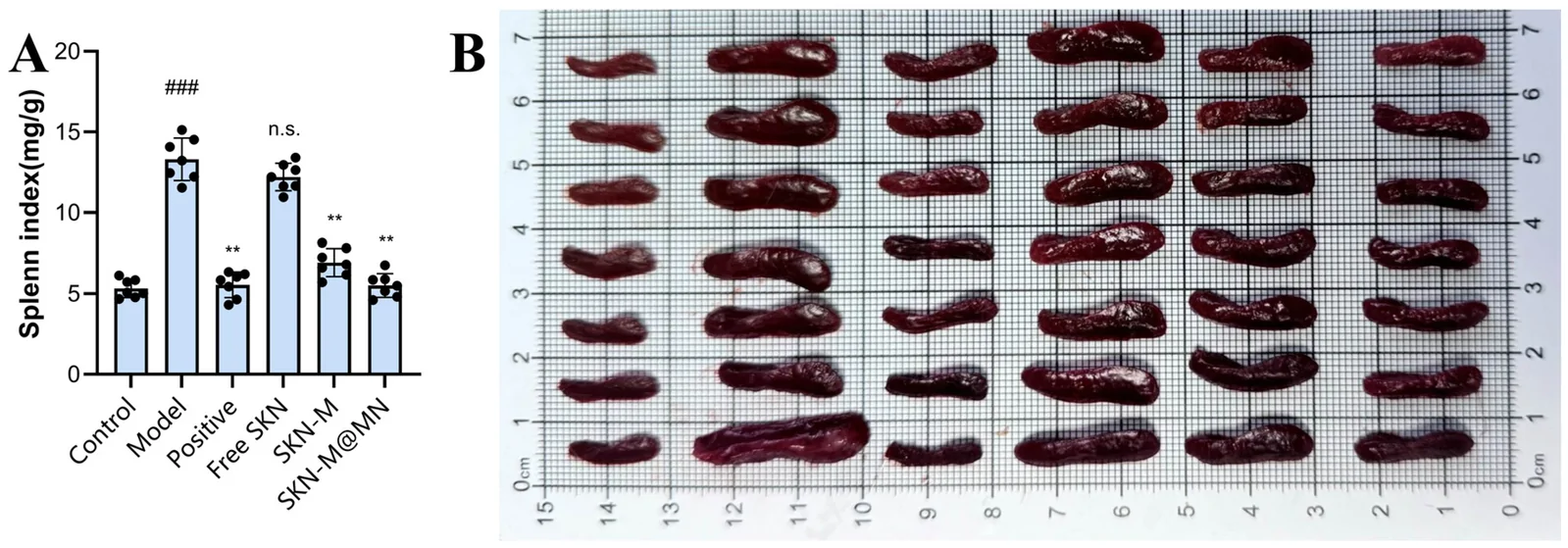
(**A**) Histogram of spleen index of mice in each group and (**B**) photograph records of spleen organs of mice in each group (*n* = 6). Note: ### *p* < 0.001 for vs. Control group. ** *p* < 0.01, vs. Model group, n.s. indicates not significant (*p* > 0.05).

**Figure 10:**
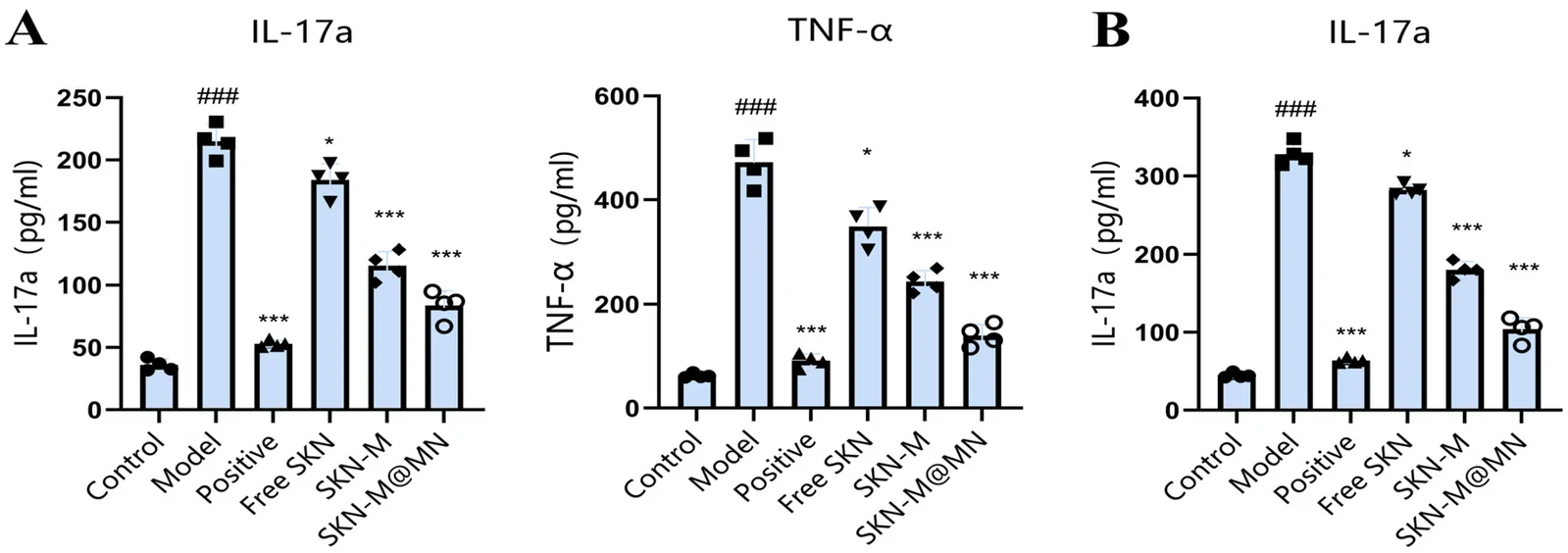
(**A**) Detection of IL-17A and TNF-α levels in serum and (**B**) skin tissue of rats in different model groups. Note: ### *p* < 0.001 vs. Control group. * *p* < 0.05, *** *p* < 0.001 vs. Model group.

**Figure 11:**
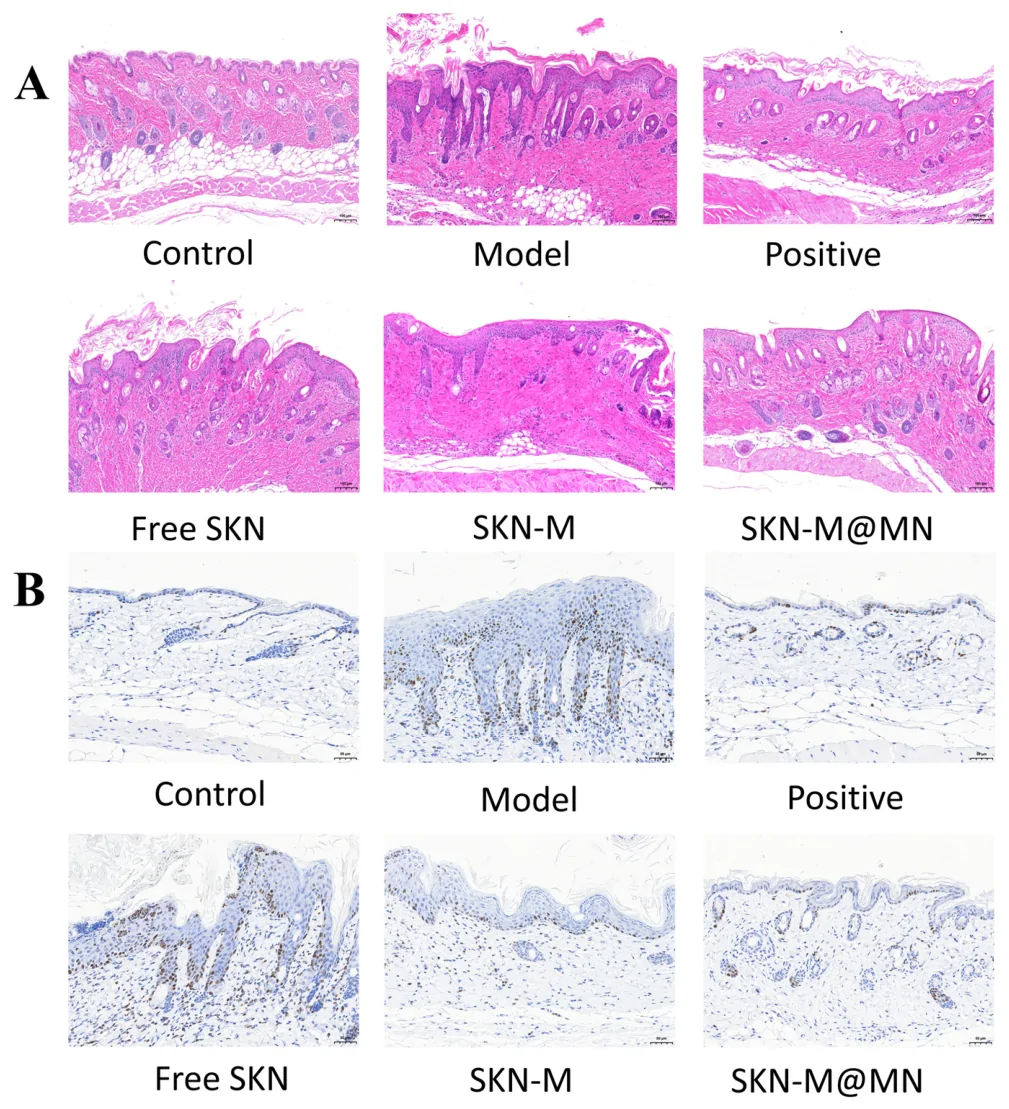
Histological and immunohistochemical analysis of skin sections from each group (Control, Model, Positive, Free SKN, SKN-M, SKN-M@MN). (**A**) H&E staining showing skin morphology and epidermal thickness (×40). Nuclei are stained in blue-purple (hematoxylin), and cytoplasm/extracellular matrix is stained in pink (eosin). (**B**) Immunohistochemical staining of Ki67, a marker of cell proliferation, in skin sections (×40). Positive Ki67 staining appears as brown (DAB chromogen), and counterstaining of nuclei appears as blue (hematoxylin).

SKN-M@MN treatment significantly reduced the spleen index to near the control level, whereas Free SKN had little effect (Figure 9A,B). Consistently, IL-17A and TNF-α levels in both serum and skin were significantly reduced by SKN-M@MN but not by Free SKN (Figure 10A,B). Histologically, H&E staining (Figure 11A) and Ki67 immunohistochemical staining (Figure 11B) showed that SKN-M@MN markedly reduced epidermal thickening and keratinocyte proliferation compared with the model group. These results indicate that SKN-M@MN alleviates psoriatic inflammation both locally and systemically.

**Figure 12:**
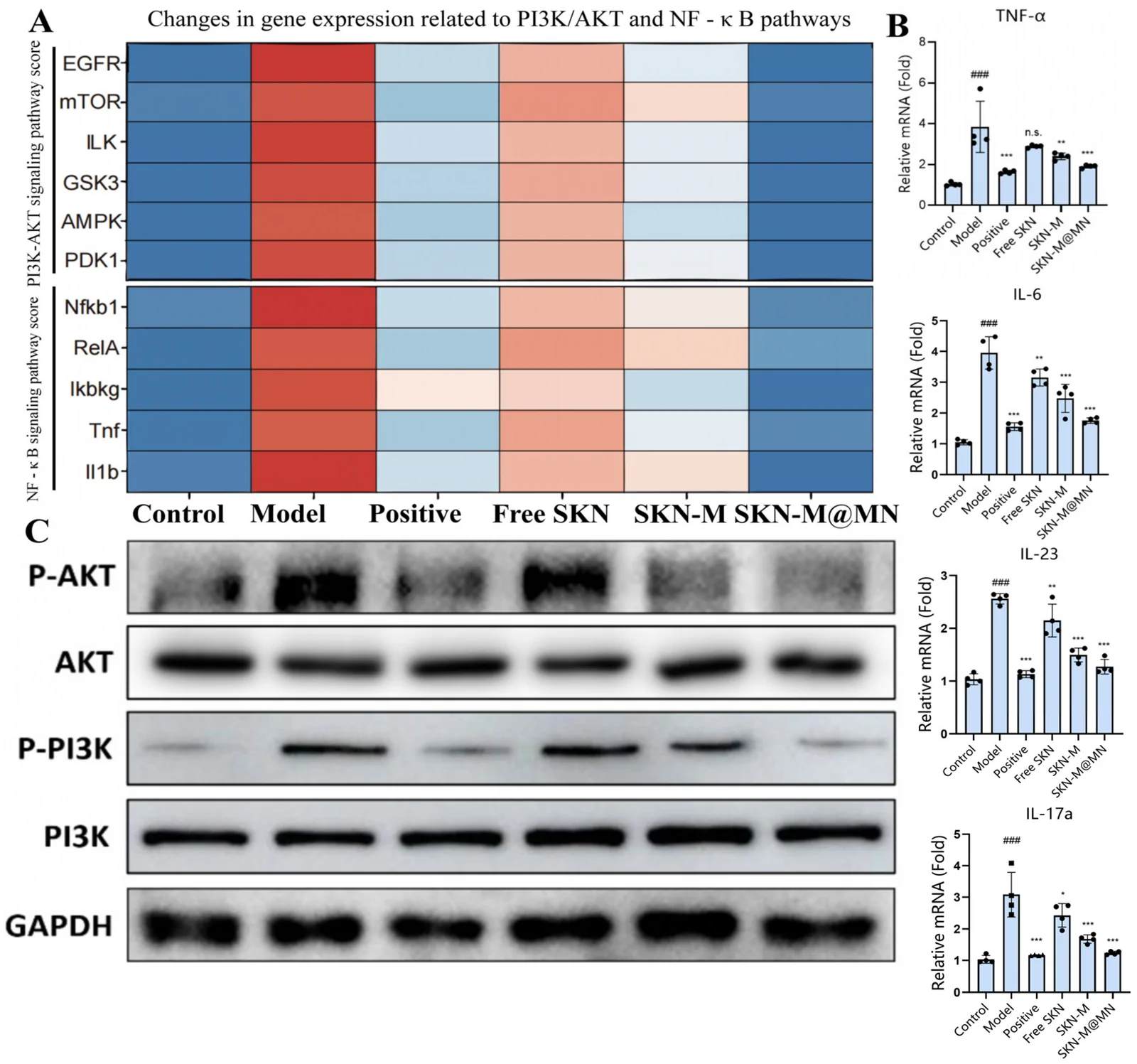
SKN-M@MN inhibits PI3K/AKT and NF-κB inflammatory signalling pathways in psoriatic rat skin. (**A**) Heatmap of RNA-seq transcriptomic data displaying differential expression of core genes involved in PI3K/AKT and NF-κB signalling across Control, Model, Positive, Free SKN, SKN-M and SKN-M@MN groups. Red represents upregulated gene expression, and blue represents downregulated gene expression. (**B**) RT-qPCR quantitative analysis of pro-inflammatory cytokines TNF-α, IL-6, IL-23 and IL-17A mRNA expression in rat skin tissues from each experimental group. (**C**) Western blot representative bands showing the protein levels of phosphorylated PI3K (P-PI3K), total PI3K, phosphorylated AKT (P-AKT) and total AKT in different groups. Note: ### *p* < 0.001 vs. Control group. * *p* < 0.05, ** *p* < 0.01, *** *p* < 0.001 vs. Model group; n.s. indicates not significant (*p* > 0.05).

RNA-seq analysis revealed that SKN-M@MN downregulated multiple genes associated with the PI3K/AKT and NF-κB signalling pathways (Figure 12A). RT-qPCR consistently showed that SKN-M@MN significantly decreased the mRNA levels of TNF-α, IL-6, IL-23 and IL-17A compared with the model group, with efficacy comparable to that of the positive control (Figure 12B).

Western blotting further demonstrated reduced protein expression of key components of the PI3K/AKT pathway in the SKN-M@MN group (Figure 12C). Collectively, these results indicate that SKN-M@MN suppresses psoriasis-related inflammation through inhibition of the PI3K/AKT and NF-κB signalling pathways.

## 4. Discussion

Psoriasis is a chronic, recurrent inflammatory skin disorder characterised by abnormal keratinocyte proliferation, immune cell infiltration, and significantly elevated reactive oxygen species (ROS) levels in the lesional microenvironment [40,41,42]. Excessively accumulated ROS not only directly damage keratinocytes but also activate downstream signalling pathways such as NF-κB and PI3K/AKT, inducing the massive secretion of pro-inflammatory cytokines including IL-17A and TNF-α, which in turn further exacerbate abnormal keratinocyte proliferation [43,44]. Currently, the commonly used clinical drugs for psoriasis-glucocorticoids, methotrexate and biologics—target different nodes in the pathological cascade, yet each possesses inherent limitations [45,46]. Topical formulations such as ointments and creams are restricted by the thickened stratum corneum barrier, resulting in low cutaneous drug bioavailability; systemic administration, although capable of elevating blood drug levels, is associated with hepatotoxicity and systemic immunosuppression [47,48,49]. Even the use of advanced nanocarriers alone struggles to penetrate the hyperkeratotic skin barrier of psoriatic lesions [50,51]. Therefore, the development of a drug delivery system that can both physically breach the stratum corneum barrier and achieve responsive drug release based on the lesional microenvironment is of significant research value.

In this study, we constructed a reactive oxygen species (ROS)-responsive hydrogel microneedle system encapsulating shikonin-loaded polymeric micelles (SKN-M@MN) to address the above-mentioned drug delivery challenges. The design rationale integrates three complementary functions: (1) Soluplus/TPGS polymeric micelles solubilise the poorly water-soluble shikonin while enhancing cellular uptake efficiency; (2) the microneedles physically penetrate the thickened stratum corneum, creating direct delivery channels for the drug to reach the lesion; (3) the HA-PBA matrix confers ROS-sensitivity: the high ROS concentration in psoriatic lesions cleaves the phenylboronate ester bonds, leading to on-demand matrix degradation and accelerated drug release. This multi-layered design simultaneously tackles the three major obstacles of poor drug solubility, skin barrier blockade, and microenvironment-specific release, which cannot be achieved by conventional single-agent formulations or non-responsive microneedles.

The optimisation results confirmed the feasibility of this composite delivery system. SKN-M exhibited an entrapment efficiency of 93.45%, a particle size of approximately 62 nm, and excellent colloidal stability, facilitating uniform loading into the microneedle tip matrix. Critically, the micelles retained their nanospherical structure after lyophilisation and reconstitution, demonstrating that the microneedle fabrication process does not compromise the integrity of the nanocarriers. At a tip-matrix-to-lyophilised-powder mass ratio of 1:2.5, SKN-M@MN achieved optimal mechanical strength and penetration performance, with 100% skin penetration. In vitro release studies showed that compared with free shikonin, SKN-M exhibited first-order sustained release kinetics, significantly mitigating the initial burst release. In a high-ROS environment (1.0 mmol/L H_2_O_2_), the HA-PBA matrix underwent accelerated degradation, leading to a markedly increased release rate of shikonin, a phenomenon consistent with the oxidative cleavage mechanism of phenylboronate esters. Franz diffusion cell permeation experiments demonstrated that, owing to the synergistic effect of microneedle-created microchannels and the enhanced diffusivity of nanoscale micelles, the SKN-M@MN group achieved the highest cumulative drug permeation among all experimental groups. Cellular uptake experiments further confirmed that the micelle formulations substantially improved the internalisation efficiency of the drug into HaCaT keratinocytes.

In an IMQ-induced psoriasis-like mouse model, SKN-M@MN significantly ameliorated erythema, scaling and epidermal thickening, with a reduction in PASI score comparable to that of the positive control (dexamethasone). Compared with free shikonin or the non-microneedle SKN-M group, SKN-M@MN exhibited superior efficacy, underscoring that microneedle-assisted permeation coupled with ROS-triggered release is the key to overcoming the pathological skin barrier. Moreover, SKN-M@MN reduced the spleen index and downregulated the levels of IL-17A and TNF-α both locally (skin) and systemically (serum), alleviating the systemic immune hyperactivation induced by psoriasis. H&E staining and Ki67 immunohistochemistry showed that the microneedle formulation effectively reversed epidermal hyperplasia and suppressed excessive keratinocyte proliferation. At the mechanistic level, transcriptome sequencing (RNA-seq), RT-qPCR and Western blotting demonstrated that SKN-M@MN downregulated the expression of genes and proteins associated with the PI3K/AKT and NF-κB pathways. Given that these two pathways are central to the regulation of keratinocyte survival, proliferation and inflammatory responses in psoriasis, our results indicate that the anti-psoriatic efficacy of SKN-M@MN is at least partially mediated through inhibition of these pathways.

Despite the notable advantages of this formulation, several limitations must be acknowledged. First, the in vitro ROS-responsiveness test used only hydrogen peroxide (H_2_O_2_) as the stimulus, whereas the psoriatic microenvironment contains a complex mixture of ROS (e.g., superoxide anion, peroxynitrite). Future studies should employ multi-component ROS cocktails for a more physiologically relevant assessment. Second, the in vitro permeation study utilised healthy rat skin, which does not fully recapitulate the hyperkeratotic barrier structure of psoriatic skin. Subsequent work could employ patient-derived ex vivo skin or reconstructed artificial epidermal models to better simulate the pathological barrier. Third, this study evaluated only short-term efficacy (7 days); long-term safety, skin irritation upon repeated administration, and in vivo pharmacokinetics of the formulation have not yet been assessed. Fourth, although we have identified PI3K/AKT and NF-κB as key pathways, the precise molecular targets of shikonin within keratinocytes and immune cells remain to be elucidated, and advanced techniques such as single-cell transcriptomics or spatial transcriptomics are warranted for deeper exploration.

In summary, the SKN-M@MN microneedle platform holds promising potential for clinical translation. Future work may include large-animal studies, optimisation of formulation storage stability, and extension of this delivery strategy to other poorly soluble anti-inflammatory natural products for the treatment of various inflammatory skin diseases.

## 5. Conclusions

In this study, an ROS-responsive hydrogel microneedle system loaded with shikonin-loaded mixed micelles (SKN-M@MN) was successfully constructed. Through the synergistic effects of microneedle-mediated physical permeation, ROS-responsive drug release and nanomicellar solubilisation, this delivery system significantly ameliorated skin lesions, inhibited the PI3K/AKT and NF-κB signalling pathways, downregulated the expression of IL-17A and TNF-α, and reversed abnormal epidermal proliferation in an IMQ-induced mouse model of psoriasis. SKN-M@MN provides an effective new strategy for the topical transdermal treatment of psoriasis with poorly soluble drugs.

## Figures and Tables

**Table 1:** Optimisation of SKN-M preparation conditions by single-factor experiments (one factor varied at a time, with others kept at baseline).

Factor	Level (Varied)	Fixed Baseline Conditions for Other Factors	Entrapment Efficiency (%)
Soluplus:TPGS (*w*/*w*)	0.5:1	Shikonin:excipients = 1:40; Temp. = 40 °C; Time = 40 min	73.64
	1:1		80.80
	**1.5:1**		92.94
	2.5:1		88.20
	3.5:1		83.25
Shikonin:excipients (*w*/*w*)	1:20	Soluplus:TPGS = 1.5:1; Temp. = 40 °C; Time = 40 min	78.24
	1:30		82.74
	**1:40**		91.86
	1:50		84.35
	1:60		84.45
Hydration temperature (°C)	35	Soluplus:TPGS = 1.5:1; Shikonin:excipients = 1:40; Time = 40 min	86.25
	40		91.76
	**45**		92.31
	50		89.41
	55		81.55
Hydration time (min)	20	Soluplus:TPGS = 1.5:1; Shikonin:excipients = 1:40; Temp. = 40 °C	73.59
	30		78.59
	40		91.56
	**50**		93.46
	60		90.43

Note: Single-factor experiments were performed independently for each factor. The bold values indicate the optimal level for each respective factor under the baseline conditions. The optimal combination (Soluplus:TPGS = 1.5:1, shikonin:excipients = 1:40, hydration temperature = 45 °C, hydration time = 50 min) was further verified in triplicate, yielding an average EE of 93.45 ± 0.24%.

**Table 2:** Physical stability of SKN-M stored at room temperature and 0–4 °C for 14 days (*n* = 3).

Condition	Time (Day)	Particle Size (nm)	PDI
Room temperature	0	62.49 ± 0.92	0.127 ± 0.012
	14	70.24 ± 0.85	0.148 ± 0.011
0–4 °C	0	62.49 ± 0.92	0.127 ± 0.012
	14	66.89 ± 0.78	0.140 ± 0.010

**Table 3:** Kinetic model fitting results of in vitro release profiles for Free SKN and SKN-M.

Model	Equation	
	FreeSKN	SKN-M
Zero-order	Q = 34.37 + 2.37t	Q = 31.13 + 2.34t
	R^2^ = 0.7496	R^2^ = 0.8339
First-order	Q = 83.03(1 − e^−0.375t^) R^2^ = 0.9616	Q = 84.73(1 − e^−0.233t^) R^2^ = 0.9739
Higuchi	Q = 14.97t^0.5^ + 18.31	Q = 15.01t^0.5^ + 13.69
	R^2^ = 0.8929	R^2^ = 0.9235
Korsmeyer–Peppas	Q = 34.21t^0.304^	Q = 29.15t^0.343^
	R^2^ = 0.9402	R^2^ = 0.9430
Hixon-crowell	Q = 100(1 − (1 − 0.0499t)^3^)	Q = 100(1 − (1 − 0.0442t)^3^)
	R^2^ = 0.7639	R^2^ = 0.7726

Note: Q = cumulative drug release percentage; t = release time; R^2^ = coefficient of determination; five classical release kinetic models including zero-order, first-order, Higuchi, Korsmeyer–Peppas and Hixon-Crowell were applied for data fitting.

## Data Availability

Data will be made available on request.

## Supplementary Materials

The following supporting information can be downloaded at: https://www.mdpi.com/article/10.3390/pharmaceutics18080939/s1, Table S1: Evaluation of SKN-M@MN performance with different mass ratios of tip matrix to lyophilized powder (*n* = 3).

## Abbreviations

The following abbreviations are used in this manuscript:

CCK-8	Cell Counting Kit-8
CLSM	confocal laser scanning microscope
CEBPD	CCAAT enhancer binding protein delta
DAB	diaminobenzidine
DAPI	4′,6-diamidino-2-phenylindole
DLS	dynamic light scattering
EE	entrapment efficiency
ELISA	Enzyme-Linked Immunosorbent Assay
GAPDH	glyceraldehyde-3-phosphate dehydrogenase
H&E	haematoxylin and eosin
HA-PBA	phenylboronic acid-modified hyaluronic acid
HPLC	high-performance liquid chromatography
HRP	horseradish peroxidase
IL-6	Interleukin-6
IL-17A	interleukin-17A
IL-23	interleukin-23
IMQ	imiquimod
MWCO	molecular weight cut-off
NF-κB	Nuclear Factor kappa-light-chain-enhancer of activated B cells
Nrf2/HO-1	Nuclear factor erythroid-2-related factor 2/Heme oxygenase-1
PASI	Psoriasis Area and Severity Index
PBS	phosphate-buffered saline
PDI	polydispersity index
PI3K/AKT	Phosphatidylinositol 3-Kinase/Protein Kinase B
PVA	polyvinyl alcohol
PVP K90	Polyvinylpyrrolidone K90
PVDF	Polyvinylidene Fluoride
ROS	reactive oxygen species
SDS-PAGE	sodium dodecyl sulphate polyacrylamide gel electrophoresis
SEM	scanning electron microscopy
SKN	Shikonin
SKN-M	shikonin-loaded polymeric micelles
SKN-M@MN	hydrogel microneedle system encapsulating SKN-loaded polymeric micelles
TNF-α	tumour necrosis factor-α
TEM	transmission electron microscope
TPGS	D-α-tocopheryl polyethylene glycol 1000 succinate
