# Supplementary material for: Shikonin-Loaded ROS-Responsive Microneedles for Psoriasis Therapy: Formulation, Transdermal Delivery, and Mechanistic Evaluation
Source: Pharmaceutics. 2026 Jul 30;18(8):939. doi: 10.3390/pharmaceutics18080939 (PMC13516278; doi:10.3390/pharmaceutics18080939)
Supplement: Supplementary file 1 [file pharmaceutics-18-00939-s001.zip › pharmaceutics-4415317-supplementary.pdf]

Table S1. Evaluation of SKN-M@MN performance with different mass ratios of tip matrix to lyophilized powder (n=3).

Mass ratio (lyophilised powder : tip matrix)	mouldability	skin penetration rate	mechanical performance
1:1.5	100	100	100
1:2	100	100	100
1:2.5	100	100	100
1:3.5	100	80	70

Table S1 Note: Scores were assigned as follows: 100 points for 100 %, 90 points for 90 %, 80 points for 80 %, etc., based on the performance of each indicator.
